# The acute physiological and perceptual effects of recovery interval intensity during cycling-based high-intensity interval training

**DOI:** 10.1007/s00421-020-04535-x

**Published:** 2020-10-23

**Authors:** Christopher R. J. Fennell, James G. Hopker

**Affiliations:** School of Sport and Exercise Sciences, University of Kent at Medway, Medway Building, Kent, Chatham, ME4 4AG England, UK

**Keywords:** Recovery components, Recovery interval intensity, High-intensity interval training, Near-infrared spectroscopy

## Abstract

**Purpose:**

The current study sought to investigate the role of recovery intensity on the physiological and perceptual responses during cycling-based aerobic high-intensity interval training.

**Methods:**

Fourteen well-trained cyclists ($\dot{V}{\text{O}}_{{{\text{2peak}}}}$: 62 ± 9 mL kg^−1^ min^−1^) completed seven laboratory visits. At visit 1, the participants’ peak oxygen consumption ($\dot{V}{\text{O}}_{{{\text{2peak}}}}$) and lactate thresholds were determined. At visits 2–7, participants completed either a 6 × 4 min or 3 × 8 min high-intensity interval training (HIIT) protocol with one of three recovery intensity prescriptions: passive (PA) recovery, active recovery at 80% of lactate threshold (80A) or active recovery at 110% of lactate threshold (110A).

**Results:**

The time spent at > 80%, > 90% and > 95% of maximal minute power during the work intervals was significantly increased with PA recovery, when compared to both 80A and 110A, during both HIIT protocols (all *P* ≤ 0.001). However, recovery intensity had no effect on the time spent at > 90% $\dot{V}{\text{O}}_{{{\text{2peak}}}}$ (*P* = 0.11) or > 95% $\dot{V}{\text{O}}_{{{\text{2peak}}}}$ (*P* = 0.50) during the work intervals of both HIIT protocols. Session RPE was significantly higher following the 110A recovery, when compared to the PA and 80A recovery during both HIIT protocols (*P* < 0.001).

**Conclusion:**

Passive recovery facilitates a higher work interval PO and similar internal stress for a lower sRPE when compared to active recovery and therefore may be the efficacious recovery intensity prescription.

## Introduction

High-intensity interval training (HIIT) is an intermittent mode of endurance training, characterised by short high-intensity work intervals (4 s to ≥ 10 min). Its discontinuous nature, by design, allows for the accumulation of a greater amount of time exercising in the ‘red zone’ (i.e. above critical power, the lactate steady state or ≥ 90% of maximal oxygen consumption [$\dot{V}{\text{O}}_{{{\text{2max}}}}$]; Buchheit and Laursen 2013), than could be tolerated during a single bout of continuous intensity exercise (MacDougall and Sale 1981). This is important because there is strong evidence that the performance of exercise at higher intensities elicits a greater activation of signalling pathways, associated with specific molecular responses which lead to an enhancement of the adaptive phenotype (Coffey and Hawley 2007). The performance benefits of HIIT alone are particularly powerful in untrained and recreationally active individuals (Milanovic et al. 2016), whilst highly trained athletes can also further enhance endurance performance by undertaking relatively short periods of HIIT (Hawley et al. 1997; Iaia and Bangsbo 2010; Laursen 2010).

The multivariate equation of HIIT programming contains five main components: work interval intensity, work interval duration, number of work intervals, recovery interval intensity and recovery interval duration (Tschakert and Hofmann 2013). Researchers have sought to optimise HIIT protocols, placing particular focus on the work interval components as this is where the training stimulus is primarily generated (Buchheit and Laursen 2013; Tschakert and Hofmann 2013). Nevertheless, optimal work interval performance (accumulating time at effective training intensities i.e. ≥ 90% $\dot{V}{\text{O}}_{{{\text{2max}}}}$), can only be achieved if separated by a correctly programmed recovery interval (Schoenmakers et al. 2019). Therefore, understanding the effects of altering the recovery interval components on subsequent work interval performance is key when looking to programme an effective HIIT session.

There has been a sizeable amount of research focusing specifically on understanding the acute effects of recovery interval intensity during cycling-based aerobic interval training (AIT; long work intervals ≥ 1 min; Barbosa et al. 2016; Coso et al. 2010; Dorado et al. 2004; Monedero and Donne 2000; McAinch et al. 2004; Siegler et al. 2006; Stanley and Buchheit 2014). Researchers investigating recovery intensity during cycling-based AIT have tended to use time to exhaustion work intervals (Barbosa et al. 2016; Siegler et al. 2006; Dorado et al. 2004) and fixed intensity work intervals (Stanley and Buchheit 2014; Coso et al. 2010). Whilst only two have utilised self-paced fixed duration work interval prescriptions (McAinch et al. 2004; Monedero and Donne 2000), which have been suggested to be an athlete’s typical approach to HIIT training (Seiler et al. 2011). McAinch et al. (2004), required participants to complete 2 × 20-min self-paced maximal effort work intervals (i.e. isoeffort) separated by a 15-min passive (PA) recovery or active (ACT) recovery at 40% of $\dot{V}{\text{O}}_{{{\text{2peak}}}}$. They found no difference in work performed during intervals between the ACT and PA protocols. Monedero and Donne (2000) used 2 × 5-km self-paced maximal effort work intervals separated by either a 20-min PA recovery, a massage, ACT recovery at 50% of $\dot{V}{\text{O}}_{{{\text{2max}}}}$, or a combined ACT recovery/massage. The combined recovery condition was found to be the most effective for maintenance of 5-km performance time. Both studies provide informative insights into the effect of recovery intensity on the performance of high-intensity AIT. However, further research utilising different HIIT protocol designs and recovery intensities is required in order to broaden the understanding of the role of recovery interval intensity on the acute responses to self-paced AIT. The current study therefore sought to investigate the role of recovery intensity on the physiological and perceptual responses during cycling-based AIT.

## Methods

### Participants

Fourteen trained cyclists participated in the study. All participants had a minimum of 2 years competitive racing experience and were in training for the next competitive season. According to De Pauw et al. (2013), participants were classified as follows: nine were performance level 3 (trained), four were performance level 4 (highly trained) and one was performance level 5 (professional). The study was completed with full ethical approval, according to the Declaration of Helsinki standards. All participants provided signed informed consent prior to testing,

### Study design

Each participant completed seven visits to the laboratory. Visit 1 being incremental exercise tests to identify the lactate threshold (LT), $\dot{V}{\text{O}}_{{{\text{2max}}}}$ and to familiarise the participants with the laboratory environment and equipment. In visits 2–7, participants performed six HIIT sessions in a randomised order (using simple randomisation; Roberts and Torgerson 1998) using different recovery intensities: PA, ACT at 80% of power output (PO) at the LT (80A) and ACT at 110% of PO at the LT (110A). The 80A and 110A recovery intensities were selected to straddle the LT and intended to provide differing levels of recovery. The 4-min and 8-min work durations were selected having previously been used in HIIT research to bring about training adaptation (Stepto et al. 1999; Seiler et al. 2011).

Visits were conducted on non-concurrent days and participants were instructed to refrain from any exercise in the day prior to testing and intense exercise in 2 days prior. Participants were instructed to arrive euhydrated and in a post-prandial state, having eaten at least 4-h prior to testing. Participants were told to not consume caffeine within 4-h and alcohol within 24-h of testing. Each participant completed all their visits to the laboratory at the same time of day to avoid any circadian variance. An electric fan was placed 2 m in front of the participants to provide cooling during all tests.

Participants used their own bike at all visits, affixed to a Cyclus2 ergometer (PO ± 2% maximal error; Rodger et al. 2016) calibrated to the manufacturer’s instructions (Leipzig, Germany). At all visits respiratory gas exchange data were assessed using breath by breath gas analysis (Metalyzer 3B; CORTEX Biophysik GmbH, Leipzig, Germany). Prior to all testing, the analyser was calibrated according to the manufacturer recommendations. Heart rate (HR) was assessed at all visits using Garmin HR monitors (Garmin, Kansas, USA).

### Preliminary testing

Participants were measured for anthropometric values: height and mass. Prior to starting the LT test resting blood lactate (B[La]) samples were taken. The participants then completed a 10-min warm-up at 50 W followed by an incremental exercise test during which PO was initially set at 80 W for 4 min, and then increased by 20 W every 4 min. The 4-min increments continued until B[La] > 4 mmol L^−1^. Participants completed a cool down for 10 min at 50 W, after which they completed seated rest for 10 min, before commencing the $\dot{V}{\text{O}}_{{{\text{2max}}}}$ test protocol.

During the LT test B[La], samples were collected using fingertip capillary blood 30 s before the end of each stage. Blood samples were analysed using a Biosen C-Line (EKF Diagnostic, London, UK). PO and HR were continuously measured throughout the test, and rating of perceived exertion (RPE) measurements were asked at the end of each stage using the Borg 6 to 20-point scale (Borg 1982). The first LT was assessed as the point at which B[La] breaks from linearity (Yoshida et al. 1987). The lactate turnpoint (LTP) was assessed as the second break point after which B[La] begins to rise above 4 mmol L^−1^ (Faude et al. 2009).

The $\dot{V}{\text{O}}_{{{\text{2max}}}}$ test protocol started with a 10-min warm-up at 100 W, after which the required cycling PO was increased by 20 W every 1 min until the participant reached volitional exhaustion (operationally defined as a cadence of < 60 revolutions/min for > 5 s, despite strong verbal encouragement). PO and HR were measured continuously throughout the test, with RPE measurements taken in the last 10 s of each 1-min stage of the test (Borg 1982). The participant’s $\dot{V}{\text{O}}_{{{\text{2peak}}}}$ was assessed as the highest pulmonary oxygen consumption ($\dot{V}{\text{O}}_{{2}}$) that was attained during a 1-min period in the test. Maximal minute power (MMP) and maximal minute heart rate (HR_max_) were assessed as the highest mean 1-min PO and HR achieved during the test.

### HIIT sessions

Participants completed both the 6 × 4-min and 3 × 8-min HIIT sessions three times (6 HIIT sessions in total), once with each of the three recovery interval intensities: PA, 80A and 110A. The ACT recovery intensities were calculated as 80% and 110% of the participants PO at the LT (Table 1). During the PA recovery intensity, HIIT session participants were instructed to remain seated with their right leg at the bottom of the pedal stroke.

**Table 1:** Participants characteristics and preliminary test results (mean ± SD)

Age (years)	33 ± 13
Height (cm)	176.6 ± 5.9
Mass (kg)	70.6 ± 8.1
VL skin fold (mm)	9.5 ± 2.7
$\dot{V}{\text{O}}_{{{\text{2peak}}}}$ (L min^−1^)	4.3 ± 0.6
Relative $\dot{V}{\text{O}}_{{{\text{2peak}}}}$ (mL kg min^−1^)	62 ± 9
MMP (W)	370 ± 56
Relative MMP (W kg^−1^)	5.2 ± 0.8
HR_max_ (bpm)	187 ± 11
PO at LT (W)	205 ± 44
PO at LTP (W)	273 ± 48
RPE at LT (6–20)	11 ± 1
RPE at LTP (6–20)	15 ± 1
80A recovery intensity (W)	164 ± 35
110A recovery intensity (W)	225 ± 48
Years training	6.8 ± 6
Years competing	6.3 ± 5.4
Mean weekly training hours	9.1 ± 2.9

*PO* power output, *LT* lactate threshold, *LTP* lactate turnpoint, *VL* vastus lateralis muscle, $\dot{V}{\text{O}}_{{{\text{2peak}}}}$ maximal oxygen consumption, *MMP* maximal minute power, *HR*_*max*_ maximal minute heart rate

All HIIT sessions had an equal work duration of 24 min. Work intervals were prescribed as self-paced on a ‘maximal session effort’ basis, with participants instructed to achieve the highest PO possible during each interval. Participants were only shown time elapsed during the HIIT sessions. Consistent verbal encouragement was given throughout every session. HIIT sessions commenced with a 10-min warm-up at 100 W and finished with a 10-min cool down at 100 W. Recovery interval durations were a standardised 2:1 work:recovery ratio (2 min and 4 min for the 6 × 4-min and 3 × 8-min HIIT sessions, respectively).

PO, HR, near-infrared spectroscopy (NIRS) and respiratory gas data were measured continuously throughout the HIIT sessions. B[La] was measured via a fingertip capillary blood sample and analysed as outlined above. Samples were taken prior to the warm-up and during the last 30 s of each work interval. RPE measurements were taken during the last 15 s of each work interval (Borg 1982). Session RPE (sRPE) measurements were taken using a 0 to 10-point scale at the end of the 10-min cool down (Foster et al. 2001).

NIRS data were acquisitioned at 10 Hz from the right vastus lateralis muscle (VL; 8 cm from the knee joint on the vertical axis) using a portable continuous-wave NIRS device (Portamon, Artinis Medical Systems, The Netherlands), which simultaneously uses the Beer-Lambert and spatially resolved spectroscopy method. Changes in tissue oxyhaemoglobin (O_2_Hb) and deoxyhaemoglobin (HHb) were measured using the differences in absorption characteristics at three wavelengths 770, 850 and 905 nm (corresponding to the absorption wavelengths of O_2_Hb and HHb). An ischemic calibration procedure was performed before each session to scale the NIRS O_2_Hb and HHb signals to the maximal physiological range, as previously described by Ryan et al. (2013). Skinfold thickness at the site of application of the NIRS optode was determined before each HIIT sessions using Harpenden skinfold callipers (British indicators Ltd, Burgess Hill, UK).

### Data analyses

Time above percentages of MMP, HR_max_ and $\dot{V}{\text{O}}_{{{\text{2peak}}}}$ during the work intervals was calculated by summing all raw PO, HR and $\dot{V}{\text{O}}_{{2}}$ measures over the established cut off. Raw PO, HR and $\dot{V}{\text{O}}_{{2}}$ data were averaged over each work and recovery interval. The Δ O_2_Hb and Δ tissue saturation index (TSI%) were calculated as the change from the last 30-s average of the work interval to the last 30-s average of the recovery interval.

### Statistical analyses

Data were presented as individual values or mean ± SD (unless specified otherwise). Statistical analyses were conducted using IBM SPSS Statistics 26 (IBM, Armonk, New York, USA). Visual inspection of Q–Q plots and Shapiro–Wilk statistics were used to check whether data were normally distributed. Three separate two-way repeated measures ANOVA, (1) two HIIT protocols (6 × 4 min vs 3 × 8 min) × three recovery intensities (PA, 80A and 110A); (2) three recovery intensities (PA, 80A and 110A) × number of work intervals; (3) three recovery intensities (PA, 80A and 110A) × number of recovery intervals were used to determine between and within condition effects for all dependent variables. Bonferroni post hoc comparisons were used when a main effect or interaction was significant. Partial eta squared (*η*_*p*_^2^) was computed as effect size estimates and were defined as small (*η*_*p*_^2^ = 0.01), medium (*η*_*p*_^2^ = 0.06) and large (*η*_*p*_^2^ = 0.14; Lakens 2013). The significance level was set at *P* < 0.05 in all cases.

## Results

Participants’ characteristics/anthropometrics are presented in Table 1.

The PA recovery protocol resulted in a longer time spent at > 80% MMP (*P* ≤ 0.001; *η*_*p*_^2^ = 0.54), > 90% MMP (*P* ≤ 0.001; *η*_*p*_^2^ = 0.62) and > 95% MMP (*P* ≤ 0.001; *η*_*p*_^2^ = 0.49) during the work intervals, when compared to the 80A and 110A recovery protocols of the 6 × 4-min and 3 × 8-min HIIT sessions. Despite the differences in time spent at high percentages of MMP, there was no effect of recovery intensity on the time spent at > 80% $\dot{V}{\text{O}}_{{{\text{2peak}}}}$ (*P* = 0.10; *η*_*p*_^2^ = 0.15), > 90% $\dot{V}{\text{O}}_{{{\text{2peak}}}}$ (*P* = 0.11; *η*_*p*_^2^ = 0.16) and > 95% $\dot{V}{\text{O}}_{{{\text{2peak}}}}$ (*P* = 0.50; *η*_*p*_^2^ = 0.05) during the work intervals of the 6 × 4-min and 3 × 8-min HIIT sessions (Table 2).

**Table 2:** Time spent in seconds above percentages of $\dot{V}{\text{O}}_{{{\text{2peak}}}}$, HR_max_ and MMP during the work intervals

Prescription	Time at %$\dot{V}{\text{O}}_{{{\text{2peak}}}}$			Time at %HR_max_			Time at %MMP		
	80	90	95	80	90	95	80	90	95
PA 6 × 4	1168 ± 141	806 ± 266	516 ± 263	1265 ± 63	954 ± 145	591 ± 221 Ωβ	940 ± 386 Ωβ	89 ± 76 Ωβ	52 ± 50 Ωβ
80A 6 × 4	1034 ± 358	669 ± 392	444 ± 328	1272 ± 96	734 ± 267	254 ± 251	625 ± 506	19 ± 28	15 ± 25
110A 6 × 4	1161 ± 372	749 ± 417	523 ± 384	1327 ± 99	902 ± 165	333 ± 236	465 ± 470	26 ± 32	15 ± 23
PA 3 × 8	1217 ± 131	841 ± 321	499 ± 301	1313 ± 59	962 ± 218 β	539 ± 268	654 ± 372 Ωβ	48 ± 39 Ωβ	27 ± 29 Ωβ
80A 3 × 8	1116 ± 334	686 ± 320	383 ± 274	1301 ± 84	817 ± 299	363 ± 288	362 ± 362	19 ± 28	14 ± 24
110A 3 × 8	1101 ± 323	640 ± 373	377 ± 332	1337 ± 54	887 ± 215	350 ± 220	209 ± 215	17 ± 25	10 ± 14

$\dot{V}{\text{O}}_{{{\text{2peak}}}}$ peak oxygen consumption, *HR*_*max*_ maximal minute heart rate, *MMP* maximal minute power, *Ω* significant difference between PA and 110A, *β* significant difference between PA and 80A, *α* significant difference between 80 and 110A

There was no effect of recovery intensity on the time spent at > 90% HR_max_ during the work intervals of the 6 × 4-min HIIT session (*P* = 0.07; *η*_*p*_^2^ = 0.42). The PA recovery protocol did increase the time spent at > 95% HR_max_ (*P* ≤ 0.001; *η*_*p*_^2^ = 0.53) during the work intervals, when compared to the 80A and 110A recovery protocols of the 6 × 4-min HIIT session. The PA recovery protocol increased the time spent at > 90% HR_max_ (*P* = 0.012; *η*_*p*_^2^ = 0.52) during the work intervals of the 3 × 8-min HIIT session, when compared to the 80A recovery protocol (*P* = 0.12) but not the 110A recovery protocol (*P* > 0.05). There was no effect of recovery intensity on the time spent at > 95% HR_max_ during the work intervals of the 3 × 8-min HIIT session (*P* = 0.10; *η*_*p*_^2^ = 0.32; Table 2).

Recovery intensity had an effect on perceptual responses with participants reporting a higher sRPE during the 110A recovery protocol, when compared to the PA and 80A recovery protocols of the 6 × 4-min HIIT session (PA, 8.3 ± 0.7 vs 80A, 8.7 ± 0.6 vs 110A, 9.1 ± 0.5 [95% CL: PA, 7.9–8.6 vs 80A, 8.3–9.0 vs 110A, 8.8–9.4]; *P* ≤ 0.001; *η*_*p*_^2^ = 0.81) and the 3 × 8-min HIIT session (PA, 8.6 ± 0.7 vs 80A, 8.5 ± 0.6 vs 110A, 9.1 ± 0.5 [95% CL: PA, 8.2–9.0 vs 80A, 8.1–8.8 vs 110A, 8.8–9.4]; *P* ≤ 0.001; *η*_*p*_^2^ = 0.79).

**Fig. 1:**
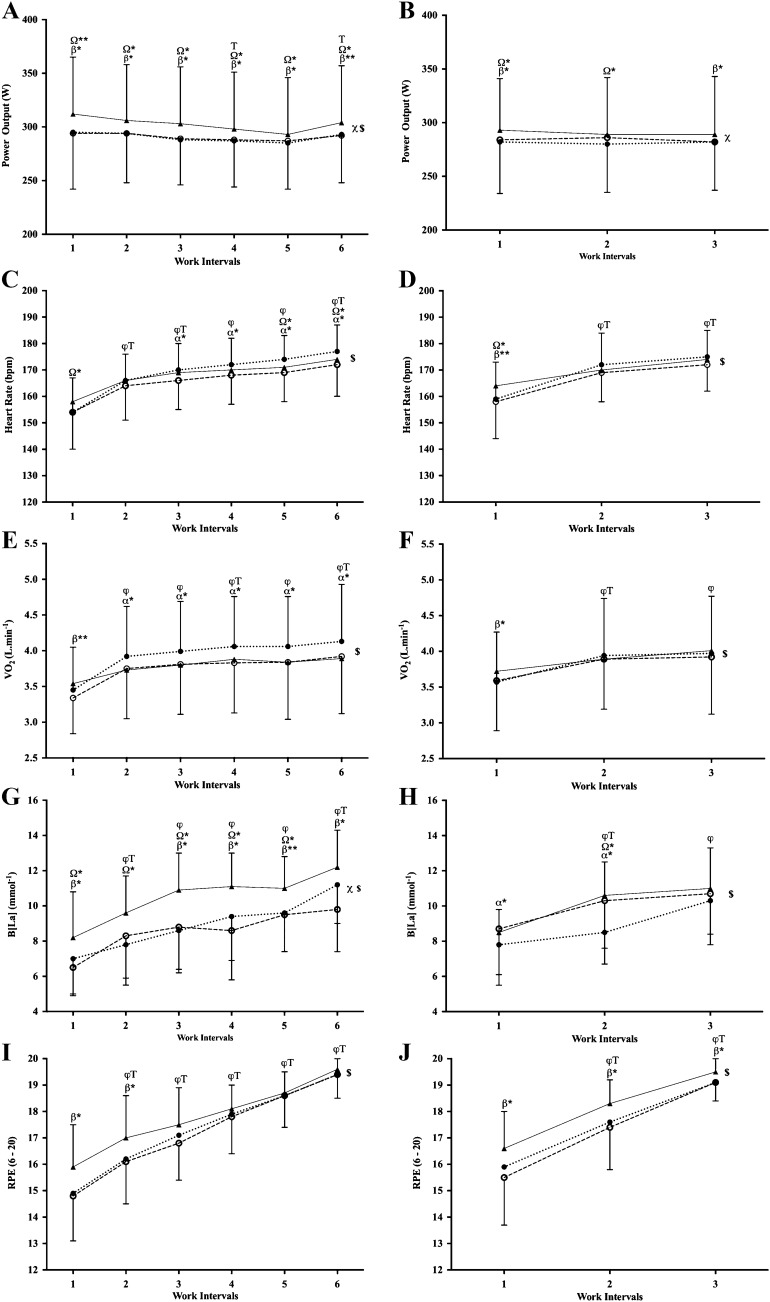
**a**, **b** Mean PO, **c**, **d** mean HR, **e**, **f** mean $$\dot{V}{\text{O}}_{{2}}$$, **g**, **h** B[La], **i**, **j** RPE. Data are displayed per work interval as mean ± SD for the 6 × 4-min and 3 × 8-min HIIT sessions with PA recovery intensity (closed triangles), 80A recovery intensity (open circles) and 110A recovery intensity (closed circles). *φ* significant difference from interval 1, *T* significant difference from previous interval, *Ω* significant difference between PA and 110A, *β* significant difference between PA and 80A, *α* significant difference between 80A and 110A, *χ* main effect of recovery intensity (all *P* < 0.01), *$* main effect of work interval number (all *P* < 0.01). *P < 0.05; **P < 0.001

Statistics and effect size estimations from the ANOVA for each work interval variable are shown in Table 3. There were interactions found between recovery intensity and work interval for PO (3 × 8; Fig. 1b), HR (Fig. 1c, d) and $\dot{V}{\text{O}}_{{2}}$ (Fig. 1e, f). No interactions between recovery intensity and work intervals were found for PO (6 × 4; Fig. 1a), B[La] (Fig. 1g, h) and RPE (Fig. 1i, j). There was a main effect of recovery intensity for PO and B[La] (6 × 4), but not for $\dot{V}{\text{O}}_{{2}}$, HR, B[La] (3 × 8) and RPE. There was a main effect of work interval number found for PO (6 × 4), HR, $\dot{V}{\text{O}}_{{2}}$, B[La] and RPE, but not for PO (3 × 8). A main effect of session type was only found for PO. Higher work interval PO was produced during the 6 × 4-min HIIT sessions, when compared to the 3 × 8-min HIIT sessions.

**Table 3:** Statistics and effect-size estimations from analysis of variance for each work interval variable analysed

Variable	Prescription	Interaction (intensity × interval)		Main effect of recovery intensity		Main effect of work interval number		Main effect of session type (6 × 4 vs 3 × 8)	
		*P*	*η*_*p*_^2^	*P*	*η*_*p*_^2^	*P*	*η*_*p*_^2^	*P*	*η*_*p*_^2^
PO	6 × 4	0.11	0.11	0.001*	0.44	0.001*	0.26	< 0.001*	0.68
	3 × 8	0.04*	0.17	0.021*	0.26	0.69	0.03		
HR	6 × 4	< 0.001*	0.43	0.09	0.19	< 0.001*	0.89	0.21	0.14
	3 × 8	< 0.001*	0.50	0.10	0.17	< 0.001*	0.83		
$\dot{V}{\text{O}}_{{2}}$	6 × 4	< 0.001*	0.32	0.06	0.20	< 0.001*	0.72	0.84	< 0.01
	3 × 8	0.006*	0.24	0.52	0.05	< 0.001*	0.74		
B[La]	6 × 4	0.08	0.12	< 0.001*	0.49	< 0.001*	0.59	0.26	0.10
	3 × 8	0.10	0.15	0.06	0.22	< 0.001*	0.53		
RPE	6 × 4	0.06	0.12	0.09	0.17	< 0.001*	0.87	0.24	0.11
	3 × 8	0.19	0.11	0.02	0.26	< 0.001*	0.86		

*PO* power output, *HR* heart rate, $\dot{V}{\text{O}}_{{2}}$ oxygen consumption, *B[La]* blood lactate concentration, *RPE* rating of perceived exertion

*Statistical significance. Effect sizes defined as small (*η*_*p*_^2^ = 0.01), medium (*η*_*p*_^2^ = 0.06), and large (*η*_*p*_^2^ = 0.14)

Recovery intensity had an effect on the physiological response of the recovery intervals. Both ACT recovery protocols produced significantly higher mean recovery interval HR (6 × 4-min: PA, 145 ± 8 vs 80A, 157 ± 11 vs 110A, 164 ± 9 bpm; 3 × 8-min: PA, 128 ± 10 vs 80A, 148 ± 11 vs 110A, 161 ± 12 bpm; *P* ≤ 0.001; *η*_*p*_^2^ = 0.89) and $\dot{V}{\text{O}}_{{2}}$ (6 × 4-min: PA, 1.9 ± 0.3 vs 80A, 3.4 ± 0.9 vs 110A, 3.8 ± 0.8 L min^−1^; 3 × 8-min: PA, 1.4 ± 0.2 vs 80A, 3.0 ± 0.6 vs 110A, 3.5 ± 0.7 L min^−1^; *P* ≤ 0.001; *η*_*p*_^2^ = 0.91) when compared to the PA protocol, during both HIIT sessions.

**Fig. 2:**
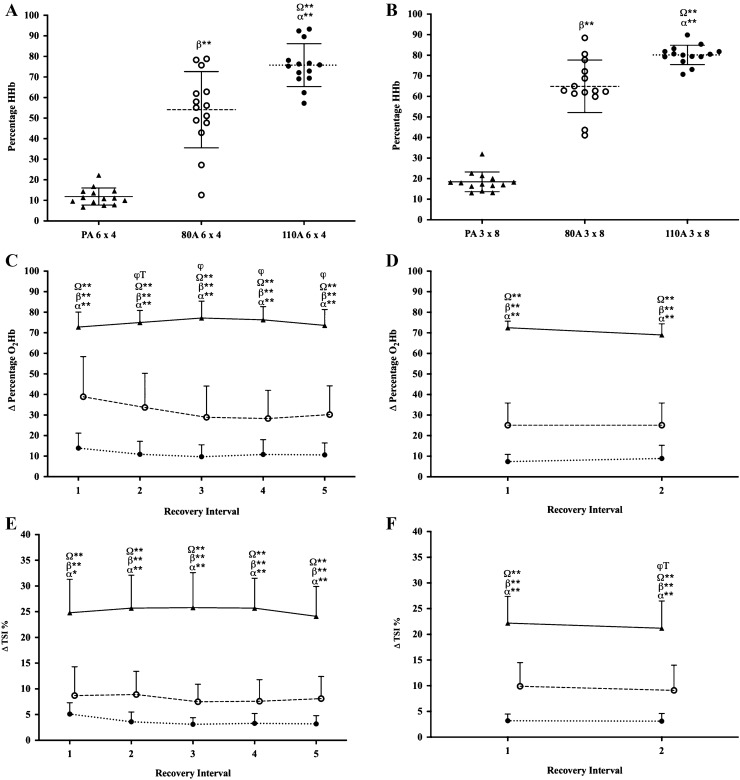
**a** Percentage HHb during the last 30 s of the recovery intervals during the 6 × 4-min HIIT sessions, **b** percentage HHb during the last 30 s of the recovery intervals during the 3 × 8-min HIIT sessions, **c** Δ O_2_Hb during the recovery intervals of the 6 × 4-min HIIT sessions, **d** Δ O_2_Hb during the recovery intervals of the 3 × 8-min HIIT sessions, **e** Δ TSI% during the recovery intervals of the 6 × 4-min HIIT sessions, **f** Δ TSI% during the recovery intervals of the 3 × 8-min HIIT sessions. PA recovery intensity (closed triangles), 80A recovery intensity (open circles) and 110A recovery intensity (closed circles). Values are mean ± SD. *φ* significant difference from interval 1, *T* significant difference from previous interval, *Ω* significant difference between PA and 110A, *β* significant difference between PA and 80A, *α* significant difference between 80A and 110A. *P < 0.05; **P < 0.001

Percentage HHb was significantly higher at the end of the recovery intervals during the 80A and 110A recovery protocols, when compared to the PA recovery protocols during both HIIT sessions (*P* ≤ 0.001; *η*_*p*_^2^ = 0.95; Fig. 2a, b). There was a greater change in percentage O_2_Hb during the PA recovery intervals, when compared to the 80A and 110A recovery intervals during both HIIT sessions (*P* ≤ 0.001; *η*_*p*_^2^ = 0.95; Fig. 2c, d). There was a greater change in TSI % during the PA recovery intervals, when compared to the 80A and 110A recovery intervals during both HIIT sessions (*P* ≤ 0.001; *η*_*p*_^2^ = 0.91; Fig. 2e, f).

## Discussion

The main finding of the study was the prescription of ACT recovery intervals significantly impairs work interval performance. Specifically, mean work interval PO (Fig. 1a, b) and time spent > 80%, > 90% and 95% of MMP (Table 2) were significantly higher during the PA recovery protocols, when compared to both ACT recovery protocols. Work interval POs were significantly higher during the 6 × 4-min HIIT protocols compared to the 3 × 8-min HIIT protocols; however, the manipulation of recovery intensity resulted in similar physiological and perceptual responses during the work intervals of both HIIT protocol designs (Table 3).

The ACT recovery intervals increased the oxygen (O_2_) demand at the exercising muscle, as shown by the greater deoxygenation of the VL (Fig. 2a, b). O_2_Hb and TSI% were therefore unable to recover to the same extent by the end of the recovery interval, in comparison to the PA protocols (Fig. 2c–f). The increased deoxygenation of the VL muscle (an important locomotor muscle during cycling performance) would potentially impair key recovery processes, such as adenosine triphosphate and phosphocreatine resynthesis, and muscle lactate clearance which require the availability of O_2_ (Spencer et al. 2006). Moreover, insufficient O_2_ availability (i.e. local hypoxia) has been suggested to affect muscular performance and exaggerate the rate of development of both central and peripheral fatigue (Amann and Calbet 2008). The more complete recovery provided by the PA protocols may explain the participant’s ability to sustain higher work interval POs, compared to the ACT recovery protocols. Buchheit et al. (2009), Kriel et al. (2016) and Ohya et al. (2013) support the findings of the current study by showing the increased deoxygenation of the VL muscle during ACT recovery lead to a reduction in work interval performance.

Time spent at high percentages of $\dot{V}{\text{O}}_{{{\text{2peak}}}}$ (≥ 90% and 95%) is often used to quantify the effectiveness of a HIIT protocol (Thevenet et al. 2007; Buchheit and Laursen 2013). When exercising close to $\dot{V}{\text{O}}_{{{\text{2peak}}}}$, the O_2_ delivery and utilisation systems are maximally stressed, which has been suggested to be an effective stimulus for improving $\dot{V}{\text{O}}_{{{\text{2max}}}}$ and endurance performance (Buchheit and Laursen 2013; Midgley et al. 2006). In the current study, recovery intensity had no effect on the duration participants spent at > 90% and > 95% of $\dot{V}{\text{O}}_{{{\text{2peak}}}}$ during both HIIT sessions (Table 2), despite the PA recovery protocols significantly reducing $\dot{V}{\text{O}}_{{2}}$ at the start of subsequent work intervals. It has been suggested that commencing work intervals from a lower metabolic rate, as observed in the PA protocols, results in a higher $\dot{V}{\text{O}}_{{2}}$ amplitude and reduces the time to reach a $\dot{V}{\text{O}}_{{2}}$ plateau during subsequent work intervals (Schoenmakers and Reed 2018). In addition, the speed of $\dot{V}{\text{O}}_{{2}}$ response has been shown to be increased at higher work rates (Hill et al. 2002). Thus, the higher work interval POs and increased time spent > 90% and > 95% of MMP during the PA recovery protocols would have likely provided a more potent driver for $\dot{V}{\text{O}}_{{2}}$, in comparison to the significantly lower work intensity of the ACT recovery protocols. The combination of the aforementioned factors provides a likely explanation for the similar times spent at high percentages of $\dot{V}{\text{O}}_{{{\text{2peak}}}}$ between PA and ACT recovery protocols.

Monitoring HR during training is commonplace for coaches and athletes, whilst HR is not directly related to muscular energy turnover or systemic O_2_ demand (Buchheit et al. 2012; Wu et al. 2005), accumulated time at > 90% HR_max_ and > 95% HR_max_ has been used to quantify adaptive effects (Seiler et al. 2011). In the present study, PA recovery lowered mean work and recovery interval HR, yet increased the time spent > 90% HR_max_ by 52–220 s and > 95% HR_max_ by 176–337 s, when compared to both ACT recovery protocols (Table 2). Aligned to the $\dot{V}{\text{O}}_{{2}}$ data, it can be inferred that PA recovery results in a faster mean response time and a higher amplitude of $\dot{V}{\text{O}}_{{2}}$ and HR during subsequent work intervals, when compared to ACT recovery (performed at ≥ 80% PO at LT). It is improbable that the increase in time > 90% HR_max_ and > 95% HR_max_ would elicit a greater adaptive stimulus. Nevertheless, our findings indicate that maintaining an elevated $\dot{V}{\text{O}}_{{2}}$ and HR during recovery is not necessary for reaching high fractions of $\dot{V}{\text{O}}_{{{\text{2peak}}}}$ and HR_max_ during subsequent work intervals.

Low-intensity ACT recovery between work intervals has been shown to be more effective in the removal of B[La] than PA recovery (Bogdanis et al. 1996; Coso et al. 2010; Siegler et al. 2006; Mandroukas et al. 2011). Current data show the ACT recovery protocols resulted in lower B[La] values when compared to the PA recovery protocols, although only significant during the 6 × 4-min HIIT session (Fig. 1g, h). This is unlikely the result of the ACT recovery intervals facilitating a greater removal of B[La] when compared to the PA recovery intervals. As B[La] measurements were taken at the end of the work intervals, it is possible that the lower B[La] values were simply due to the lower work interval intensity of the ACT protocols (Fig. 1a, b). In accordance with evidence showing B[La] does not inhibit exercise performance (Hall et al. 2016), the higher B[La] values attained during the PA protocols did not affect subsequent work interval PO. These data support the prescription of PA recovery for increasing the metabolic stress during HIIT sessions, without affecting work interval performance. Whilst research should be used to guide HIIT design, coaches and athletes are advised to be cautious when extrapolating the findings beyond the scope of the HIIT protocols used.

There was a clear linear increase in work interval RPE throughout all HIIT sessions, with reported RPE values reaching ≥ 18 (very hard) at the last work interval (Fig. 1i, j). The upward drift in physiological stress throughout the HIIT sessions provides an explanation for the increase in RPE, whilst it is also highly likely that biomechanical and psychological processes also effected the participant’s RPE (Marcora et al. 2009; Ulmer 1996). The higher RPE values reported during the PA protocols maybe linked to the higher work interval POs (Fig. 1a, b) and percentages of MMP (Table 2) achieved during the PA protocols in comparison to the ACT protocols. Despite within session RPE being higher during the PA protocols, participants reported significantly higher sRPE values at the end of the 110A recovery protocol when compared to the 80A and PA recovery protocols during both HIIT sessions. This finding is of particular interest from an applied perspective when programming HIIT. A HIIT protocol design which reduces an individual’s sRPE without negatively affecting the physiological and metabolic load would likely be seen as a favourable session prescription by both athlete and coach.

## Conclusion

ACT recovery at 80% and 110% of the LT significantly impairs performance PO but has a limited effect on the physiological stress of the work intervals during two closely matched HIIT designs, when compared to PA recovery. Based on current evidence, PA recovery between long ‘aerobic’ work intervals facilitates a higher external training load whilst maintaining a similar internal stress for a lower sRPE and therefore may be the efficacious recovery intensity prescription.

## Data Availability

Data transparency.

## Abbreviations

ANOVA: Analysis of variance
ACT: Active
AIT: Aerobic interval training
B[La]: Blood lactate concentration
HR: Heart rate
HR_max_: Maximal minute heart rate
HIIT: High-intensity interval training
HHb: Deoxyhaemoglobin
LT: Lactate threshold
NIRS: Near-infrared spectroscopy
O_2_Hb: Oxyhaemoglobin
O_2_: Oxygen
PA: Passive
PO: Power output
RPE: Rating of perceived exertion
sRPE: Session RPE
TSI%: Tissue saturation index
VL: Vastus lateralis muscle
$\dot{V}{\text{O}}_{{\text{2}}}$: Pulmonary oxygen uptake
$\dot{V}{\text{O}}_{{{\text{2peak}}}}$: Peak oxygen consumption
$\dot{V}{\text{O}}_{{{\text{2max}}}}$: Maximal oxygen consumption
MMP: Maximal minute power
80A: 80% Power output at lactate threshold
110A: 110% Power output at lactate threshold

## Code availability

Data analysis software application used (SPSS) openly available.
